# An efficient unified model for genome-wide association studies and genomic selection

**DOI:** 10.1186/s12711-017-0338-x

**Published:** 2017-08-24

**Authors:** Hengde Li, Guosheng Su, Li Jiang, Zhenmin Bao

**Affiliations:** 1 Ministry of Agriculture Key Laboratory of Aquatic Genomics, CAFS Key Laboratory of Aquatic Genomics and Beijing Key Laboratory of Fishery Biotechnology, Center for Applied Aquatic Genomics, Chinese Academy of Fishery Sciences, Beijing, 100141 China; 2 Center for Quantitative Genetics and Genomics, Department of Molecular Biology and Genetics, Aarhus University, 8830 Tjele, Denmark; 3 College of Marine Life, Ocean University of China, Qingdao, 266003 China

## Abstract

**Background:**

A quantitative trait is controlled both by major variants with large genetic effects and by minor variants with small effects. Genome-wide association studies (GWAS) are an efficient approach to identify quantitative trait loci (QTL), and genomic selection (GS) with high-density single nucleotide polymorphisms (SNPs) can achieve higher accuracy of estimated breeding values than conventional best linear unbiased prediction (BLUP). GWAS and GS address different aspects of quantitative traits, but, as statistical models, they are quite similar in their description of the genetic mechanisms that underlie quantitative traits.

**Methods:**

Here, we propose a stepwise linear regression mixed model (StepLMM) to unify GWAS and GS in a single statistical model. First, the variance components of the genomic-BLUP (GBLUP) model are estimated. Then, in the SNP selection step, the linear mixed model (LMM) for GWAS is equivalently transformed into a simple linear regression to improve computation speed, and the most significant SNP is selected and included into the evaluation model. In the SNP dropping step, the SNPs in the evaluation model are tested according to the standard errors of their estimated effects. If non-significant SNPs are present, the least significant one is dropped from the model and variance components are re-estimated. We used extended Bayesian information criteria (eBIC) to evaluate the model optimization, i.e. the model with the smallest eBIC is the final one and includes only significant SNPs.

**Results:**

We simulated scenarios with different heritabilities with 100 QTL. StepLMM estimated heritability accurately and mapped QTL precisely. Genomic prediction accuracy was much higher with StepLMM than with GBLUP. The comparison of StepLMM with other GWAS and GS methods based on a dataset from the 16th QTLMAS Workshop showed that StepLMM had medium mapping power, the lowest rate of false positives for QTL mapping, and the highest accuracy for genomic prediction.

**Conclusions:**

StepLMM is a combination of GWAS and GBLUP. GWAS and GBLUP are beneficial to each other in a single statistical model, GWAS improves genomic prediction accuracy, while GBLUP increases mapping precision and decreases the rate of false positives of GWAS. StepLMM has a high performance in both GWAS and GS and is feasible for agricultural breeding programs and human genetic studies.

**Electronic supplementary material:**

The online version of this article (doi:10.1186/s12711-017-0338-x) contains supplementary material, which is available to authorized users.

## Background

The genetic mechanisms that underlie a quantitative trait are complicated processes to analyse. Under the infinitesimal model [1], it is assumed that a quantitative trait is determined by an infinite number of unlinked and non-epistatic loci, each with an infinitesimal effect, that satisfy normality and linearity. Based on the infinitesimal hypothesis, best linear unbiased prediction (BLUP) [2] is an effective method to predict genetic values based on the resemblance between genetically-related animals within a pedigree. With the advancement of high-throughput genotyping, prediction of genetic values can also be inferred from genome-wide single nucleotide polymorphism (SNP) data, referred to as genomic selection [3].

Many statistical models and algorithms are available for genomic prediction, which differ in the assumptions regarding the distribution of SNP effects. For example, Bayesian variable selection models [4] and least absolute shrinkage and selection operator (LASSO) models [5] assume that some SNPs have large or moderate effects and the others have small or null effects, while linear mixed models assume that the effects of all SNPs are normally distributed with equal variance [6]. The genomic BLUP (GBLUP) model is a linear mixed model, which integrates a genomic relationship matrix that is built using the information of SNPs, instead of a pedigree-based relationship matrix [6, 7]. This model has become a frequently used method for genomic prediction in plant and animal breeding [8–10].

A genomic estimated breeding value is obtained by summing the effects of all variants but it is generally acknowledged that, among the whole set of variants, some have a larger genetic effect on the trait of interest than others. Genome-wide association studies (GWAS) have validated the existence of causal variants and have become an important tool to identify variants that underlie human diseases and agriculturally important traits. Nevertheless, performing GWAS and quantitative trait loci (QTL) analyses at the genome-wide level is a challenging issue. Population structure or relatedness between individuals can lead to a high rate of false positives and to lower mapping precision and statistical power. The linear mixed model (LMM) is an effective method to handle population structure [11], but compared to the linear regression model (LRM), which is widely used in human genetics, LMM is computationally demanding. Although it takes only a few seconds to perform an association analysis for one variant, the total computation time necessary for millions of genomic variants is unimaginable. To improve the computation efficiency of GWAS, several methods were developed. The GRAMMAR method [12] first adjusts observations for family effects to estimate the residuals, and then analyses the association between SNPs and the residuals. To further decrease computational burden, Zhang et al. [13] compressed LMM by clustering individuals into groups and using a two-step association analysis to eliminate the need of re-computing variance components, which significantly improved computation efficiency. However, the clustering process by compression of the genetic relationship matrix eliminates the possibility of predicting genetic values, because individuals in the same group share the same genetic value. Kang et al. [14] proposed the EMMA algorithm to improve iteration speed, but solving mixed model equations (MME) with a large sample size and hundreds of thousands of variants remains computationally intensive. Meyer and Tier [15] found that the coefficient matrix ${\mathbf{C}}_{11}$ of MME and ${\mathbf{C}}_{11}^{ - 1}$ were constant across the multiple analyses for individual SNPs, and proposed a computing strategy named SNP Snappy.

The above methods are based on a single-locus model combined with a realized genetic relationship matrix to account for confounding effects. Because quantitative traits are controlled by multiple loci, a multiple-locus model may be more appropriate. It was reported that a multiple-locus model outperforms a single-locus model in traditional QTL mapping [16]. A multiple-locus model for GWAS is not only more robust than a single-locus model in the statistical sense, but it also brings more computational burden. Shrinkage is an efficient method to select latent sparse predictors from genome-wide variants. Lee et al. [17] developed a Bayesian method that uses genome-wide markers to predict phenotypes simultaneously. Li et al. [18] proposed the Bayesian Lasso model for GWAS but it ignores population structure. Rakitsch et al. [19] presented the LMM-Lasso model, which corrects confounding effects with LMM and then selects candidate variants by Lasso regression. Although LMM-Lasso is efficient in computation cost, it approximates the variance components for confounding that is caused by the genetic background of variants and random errors, and these are assumed to remain unchanged after new variants become cofactors in the statistical model for GWAS, which may have unfavourable effects on the subsequent genomic prediction. Segura et al. [20] suggested a multiple loci mixed model (MLMM) that introduced a stepwise regression with forward inclusion and then backward elimination of variants. A more general and comprehensive stepwise regression would be to include a new variant into the model conditionally on the GWAS results based on residuals of the model and to drop the least significant variant through a significance test until all variants in the model are significant and those outside of the final model are not significant, as in multiple linear regression. From a genomic prediction point of view, a combination between the linear mixed model and the sparse regression model is more accurate than either model separately [21, 22].

As an alternative, we proposed a stepwise linear mixed regression model that is stable, flexible and computationally efficient. Importantly, this model can be used both for GWAS and GS simultaneously. We used the linear mixed model and a realized relationship matrix to handle population structure or confounding effects. At each regression step, the variance components are re-estimated by an efficient mixed model (EMM) approach. Then, the linear mixed regression model is equivalently transformed into a simple linear model by removing the influence of random effects (see “Methods”), which decreases computation time significantly. For the genetic evaluation model, we used extended Bayesian information criteria (eBIC) as convergence criteria, which are useful and stringent criteria for model selection in GWAS [23]. The model for which the eBIC reaches the lowest value is considered as the final model, and all the variants that it includes are significant quantitative trait loci (QTL). We evaluated our approach and demonstrated its utility by applying it in GWAS and GS on simulated data for human genetic analyses and agricultural breeding.

## Methods

### Linear mixed model

The phenotypic value is decomposed as:

1 $${\mathbf{y}} = {\mathbf{Xb}} + {\mathbf{Zq}} + {\mathbf{Wg}} + {\mathbf{e}},$$

where ${\mathbf{y}}$ is the vector of phenotypes; ${\mathbf{b}}$ is the vector of fixed systematic effects; ${\mathbf{q}}$ is the vector of allele substitution effects of the major QTL, which are treated as fixed effects; ${\mathbf{g}}$ is the vector of additive genetic effects explained by the polygenes, ${\mathbf{g}}\,{\sim}\,N({\mathbf{0}},{\mathbf{K}}\sigma_{g}^{2})$, where ${\mathbf{K}}$ is the realized genetic relationship matrix calculated from genome-wide SNP information [6], and $\sigma_{g}^{2}$ is the genetic variance explained by the polygenes; ${\mathbf{X}}$ and ${\mathbf{W}}$ are the corresponding design matrices for ${\mathbf{b}}$ and ${\mathbf{g}}$; ${\mathbf{Z}}$ is the matrix of genotype codes for SNPs with large effects; ${\mathbf{e}}$ is the vector of residuals, and ${\mathbf{e}}\,{\sim}\,N({\mathbf{0}},{\mathbf{I}}\sigma_{e}^{2})$, where $\sigma_{e}^{2}$ is the variance of the random errors. The overall phenotypic variance–covariance matrix can be expressed as: ${\mathbf{V}} = \sigma_{g}^{2} {\mathbf{WKW}}^{ '} + \sigma_{e}^{2} {\mathbf{I}}$.

The mixed model equations for Model (1) are:

2 $$\left[ {\begin{array}{*{20}c} {{\mathbf{X}}^{{\prime }} {\mathbf{X}}} & {{\mathbf{X}}^{{\prime }} {\mathbf{Z}}} & {{\mathbf{X}}^{{\prime }} {\mathbf{W}}} \\ {{\mathbf{Z}}^{{\prime }} {\mathbf{X}}} & {{\mathbf{Z}}^{{\prime }} {\mathbf{Z}}} & {{\mathbf{Z}}^{{^{{\prime }} }} {\mathbf{W}}} \\ {{\mathbf{W}}^{{\prime }} {\mathbf{X}}} & {{\mathbf{W}}^{{\prime }} {\mathbf{Z}}} & {{\mathbf{W}}^{{\prime }} {\mathbf{W}} +\uplambda{\mathbf{K}}^{ - 1} } \\ \end{array} } \right]\left[ {\begin{array}{*{20}c} {{\hat{\mathbf{b}}}} \\ {{\hat{\mathbf{q}}}} \\ {{\hat{\mathbf{g}}}} \\ \end{array} } \right] = \left[ {\begin{array}{*{20}c} {{\mathbf{X}}^{{\prime }} {\mathbf{y}}} \\ {{\mathbf{Z}}^{{\prime }} {\mathbf{y}}} \\ {{\mathbf{W}}^{{\prime }} {\mathbf{y}}} \\ \end{array} } \right],$$

where $$\uplambda = \sigma_{e}^{2} /\sigma_{g}^{2}$$.

Model (1) can be compared with the null model:

3 $${\mathbf{y}} = {\mathbf{Xb}} + {\mathbf{Wg}} + {\mathbf{e}},$$

to test the significance of individual SNPs.

### Stepwise linear mixed model (StepLMM)

In order to improve computation efficiency, GWAS with LMM can be conducted alternatively by applying a two-stage strategy. First, variance components are estimated with LMM and ${\mathbf{V}}$ is calculated, second, both the genotype matrix and observation vector are rotated with the inverse of ${\mathbf{V}}$, and a simple regression with rotated data for individual SNPs is performed. This strategy was proven to yield near-identical results to an exact approach [24]. Likewise, the first step of StepLMM is to estimate $\sigma_{g}^{2}$ and $\sigma_{e}^{2}$ by restricted maximum likelihood (REML) under a null model that ignores the effect of individual SNPs. Because the spectral decomposition of the ${\mathbf{G}}$ matrix and the conversion of the restricted maximum likelihood into a one-dimensional optimizer can improve the computation speed of LMM [14, 25, 26], we adopted this approach to find the optimized $\sigma_{g}^{2}$ and $\sigma_{e}^{2}$. Consequently, the single-SNP association analysis can be equivalently performed with simple linear regression after transformation of ${\mathbf{y}}$ and SNP genotype code ${\mathbf{m}}$. First, we calculated matrix ${\mathbf{V}}$ with the estimated variance components and inverted it, then calculated ${\mathbf{L}}$ by Cholesky decomposition of ${\mathbf{V}}^{ - 1}$ with the equation:

4 $${\mathbf{L}}^{ '} {\mathbf{L}} = {\mathbf{V}}^{ - 1}$$

where ${\mathbf{L}}$ is an upper-triangular matrix. The model for the association analysis can be simplified as:

5 $${\mathbf{y}}^{*} = {\mathbf{X}}^{*} {\mathbf{b}}^{*} + {\mathbf{Z}}^{*} {\mathbf{q}}^{*} + {\mathbf{e}}^{*} ,$$

where

6 $${\mathbf{y}}^{*} = {\mathbf{L}} \cdot {\mathbf{y}},$$

7 $${\mathbf{X}}^{*} = {\mathbf{L}} \cdot {\mathbf{X}},$$

8 $${\mathbf{Z}}^{*} = {\mathbf{L}} \cdot {\mathbf{Z}}.$$

${\mathbf{y}}^{*}$ is the vector of transformed observation values, ${\mathbf{X}}^{*}$ is the transformed ${\mathbf{X}}$ matrix, ${\mathbf{Z}}^{*}$ is the matrix of transformed SNP genotype codes, and ${\mathbf{e}}^{*}$ is the vector of transformed random errors, respectively. ${\mathbf{b}}^{*}$ and ${\mathbf{q}}^{*}$ are the vectors of fixed non-genetic effects and SNP effects in the transformed scale, respectively. After transformation, the association test can be alternatively conducted with a linear regression model. This is more efficient for computation than LMM by avoiding the iteration process of the association test using LMM for each SNP.

For stepwise linear regression, the most significant SNP is selected for the full model by the log-likelihood ratio test based on a simplified model, and this SNP will be kept at least once. At the same time, if any insignificant SNP existed in the full model, the least significant one would be dropped according to Student’s *t* test as follows:

9 $$t = \frac{q}{{\sqrt {\sigma_{e}^{2} \cdot diag({\mathbf{C}}^{22} )} }},$$

where ${\mathbf{C}}^{22}$ is the elements corresponding to ${\mathbf{q}}$ in the inverse of the left hand side (LHS) of MME,

10 $${\mathbf{LHS}}^{ - 1} = \left[ {\begin{array}{*{20}c} {{\mathbf{C}}^{11} } & {{\mathbf{C}}^{12} } & {{\mathbf{C}}^{13} } \\ {{\mathbf{C}}^{21} } & {{\mathbf{C}}^{22} } & {{\mathbf{C}}^{23} } \\ {{\mathbf{C}}^{31} } & {{\mathbf{C}}^{32} } & {{\mathbf{C}}^{33} } \\ \end{array} } \right].$$

**Fig. 1:**
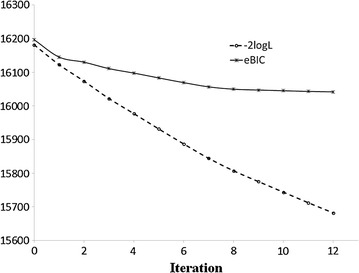
An example of the iterations of the StepLMM model. The *horizontal axis* represents the iteration round, and the *vertical axis* is the value of the extended Bayesian information criteria and −2 * log-likelihood (−2logL) of likelihood. As the iteration number increases, eBIC and −2logL decrease, and the final optimized model is achieved when the eBIC does not decrease anymore

The degree of freedom is $n_{obs} - n_{beta} - n_{qtn}$, where $n_{obs}$, $n_{beta}$ and $n_{qtn}$ are number of observation, rank of ${\mathbf{X}}$ and number of SNPs in the full model, respectively. The stepwise procedure was performed by repeating the selection and dropping. In this study, we used eBIC [23] as a measure of model-fit, which can tightly control the rate of false positives with a small loss in mapping power. When a new SNP is included, the eBIC of the model must be smaller than that of the last model without this SNP. The stepwise procedure stops when the eBIC cannot decrease anymore and all the variants in the final model are significant QTL (Fig. 1).

The algorithm can be summarized as follows:

- Step 1: estimate $\sigma_{g}^{2}$ and $\sigma_{e}^{2}$ using Model (3);
- Step 2: calculate ${\mathbf{V}}$ and decompose it with Eq. (4), then transform ${\mathbf{y}}$, ${\mathbf{X}}$ and ${\mathbf{Z}}$ with Models (6), (7), and (8), respectively;
- Step 3: perform a significance test for individual SNPs with Model (5);
- Step 4: select the new, most significant SNP in the model and estimate variance components with Model (1);
- Step 5: test the significance of old SNPs in the model with Eq. (9), and if non-significant SNPs exist, drop the one that is least significant and estimate variance components with Model (1);
- Step 6: repeat Step 2 through Step 5 until the eBIC of the model cannot decrease anymore;
- Step 7: estimate the breeding values and QTL effects.

### Evaluation of the model

The proposed StepLMM model was evaluated in terms of mapping precision and prediction accuracy. Mapping precision was evaluated by the number of QTL detected and the number of QTL located at defined regions with certain genome lengths (0, 10, 20 kb) on either side of a true QTL. Genomic prediction accuracy was defined as the correlation between genomic estimated breeding values (GEBV) and true breeding values. In the above model, an individual GEBV is: ${\mathbf{GEBV}} = {\mathbf{Zq}} + {\mathbf{Wg}}$. The variance of QTL $i$ is $\sigma_{{q_{i} }}^{2} = 2p_{i} \left( {1 - p_{i} } \right)q_{i}^{2}$, where $p_{i}$ is the minor allele frequency of QTL $i$, and the total QTL variance is $\sigma_{q}^{2} = \mathop \sum \nolimits 2p_{i} (1 - p_{i} )q_{i}^{2}$. Therefore, the heritability is calculated as $h^{2} = \frac{{\sigma_{q}^{2} + \sigma_{g}^{2} }}{{\sigma_{q}^{2} + \sigma_{g}^{2} + \sigma_{e}^{2} }}$. To evaluate our StepLMM model, we also compared it with traditional genomic selection methods such as GBLUP and BayesB.

### Data

We used Wellcome Trust Case Control Consortium (WTCCC) human genotypic data for the simulation (https://www.wtccc.org.uk/info/access_to_data_samples.html). The data consisted of 2000 samples each from the following disease collections: type 1 diabetes, type 2 diabetes, rheumatoid arthritis, inflammatory bowel disease, bipolar disorder, hypertension and coronary artery disease. The samples were genotyped with 500,568 SNPs. SNPs with a minor allele frequency (MAF) less than 0.05 were removed from the analysis. We simulated one complex trait controlled by 100 QTL. The causal variants were randomly chosen and the effect of each locus was randomly drawn from an exponential distribution with a rate of 1. The genetic value of an individual was defined as the sum of the effects of all loci, i.e., the 100 QTL accounted for the whole genetic variance. The phenotypic value was generated by adding a random residual to the genetic value, and a random residual was drawn from a normal distribution with a mean of zero and scaled variance to fix the trait heritability to 0.25, 0.50 and 0.75. For each scenario, 50 replicates were simulated to test the performance of our method.

In addition, we used QTLMAS16 datasets (http://qtl-mas-2012.kassiopeagroup.com/en/dataset.php) to compare our method with methods in the literature. The QTLMAS16 data consisted of simulated genotypes, true breeding values for three traits with different heritabilities for 4100 samples, among which 3000 samples were phenotyped as training data.

## Results

### Mapping precision

Table 1 shows the mapping precision of StepLMM. Averaged over replicates, the number of identified QTL was 7.78 for the trait with a low heritability ($h^{2}$ = 0.25) and sharply increased to 34.22 for the trait with a high heritability ($h^{2}$ = 0.75). Mapping precision also increased when heritability increased. For all scenarios in terms of heritability, more than 65% of the identified QTL were mapped exactly to their positions under all scenarios. If a SNP within a certain distance on either side of a causal SNP was considered as a QTL, then the mapping precision in the scenario of medium heritability ($h^{2}$ = 0.50) within ±10, ±20, and ±30 kb lengths were 0.83, 0.87 and 0.90, respectively. The mapping precisions for the scenarios of low and high heritability were approximately 3% lower or higher than those for the scenarios of medium heritability, respectively.

**Table 1:** Mapping precision of the stepwise linear mixed model based on WTCCC simulated data

$h^{2}$	nQTL^a^	Number of detected causal SNPs				Mapping precision			
		0 kb	10 kb	20 kb	30 kb	0 kb	10 kb	20 kb	30 kb
0.25	7.78 (0.345)	5.01 (0.171)	6.22 (0.154)	6.54 (0.154)	6.69 (0.132)	0.65 (0.022)	0.80 (0.019)	0.84 (0.019)	0.86 (0.017)
0.50	18.52 (0.522)	12.22 (0.333)	15.37 (0.222)	15.37 (0.185)	16.67 (0.167)	0.66 (0.018)	0.83 (0.012)	0.87 (0.010)	0.90 (0.009)
0.75	34.22 (0.640)	23.95 (0.376)	29.42 (0.308)	30.80 (0.240)	31.82 (0.205)	0.70 (0.011)	0.86 (0.009)	0.90 (0.007)	0.93 (0.006)

$h^{2}$: heritability of simulated traits

100 QTL were simulated for all scenarios

^a^nQTL is the number of significant QTL

Values in parentheses are the corresponding standard errors

### Genomic prediction

Using the StepLMM presented here, the estimated heritability was quite close to true heritability (Table 2). Because our method consists of both a major genetic effect (fixed effect) and a minor polygenic effect (random effect), we calculated the proportion of genetic variance explained by the identified QTL and three correlations: correlation between true breeding value (TBV) and genomic breeding value predicted by the detected QTL, correlation between TBV and random polygenic effects, and correlation between TBV and genomic estimated breeding value (GEBV). The identified QTL explained 68, 81 and 92% of the genetic variance for a trait with a low ($h^{2}$ = 0.25), medium ($h^{2}$ = 0.50) and high ($h^{2}$ = 0.75) heritability, respectively. With StepLMM, the accuracy of genomic values predicted by QTL only was also high but slightly lower than the accuracy of GEBV and higher than that of GEBV from GBLUP. Without accounting for QTL effects, the correlation between the remaining polygenic effect and the TBV was low and significantly lower than those of GBLUP.

**Table 2:** Comparison of genomic prediction accuracy between the stepwise linear mixed model (StepLMM) and genomic best linear unbiased prediction (GBLUP) based on WTCCC simulated data

$\varvec{h}^{2}$	$\widehat{\varvec{h}}^{2}$	$\widehat{\varvec{h}}_{\varvec{q}}^{2} /\widehat{\varvec{h}}^{{2^{\text{a}} }}$	$r_{qtl}^{\text{b}}$	$r_{poly}^{\text{c}}$	$r_{StepLMM}^{\text{d}}$	$r_{GBLUP}^{\text{e}}$	$b_{StepLMM}^{\text{f}}$	$b_{GBLUP}^{\text{g}}$
0.25	0.25 (0.009)	0.68 (0.026)	0.71 (0.010)	0.27 (0.009)	0.75 (0.007)	0.49 (0.008)	0.89 (0.011)	1.16 (0.055)
0.50	0.52 (0.007)	0.81 (0.013)	0.87 (0.004)	0.23 (0.006)	0.90 (0.002)	0.71 (0.002)	0.94 (0.005)	1.04 (0.042)
0.75	0.76 (0.004)	0.92 (0.007)	0.95 (0.002)	0.18 (0.007)	0.96 (0.001)	0.86 (0.001)	0.98 (0.002)	1.01 (0.022)

^a^Proportion of phenotypic variance explained by the QTL in the models, $h_{q}^{2} = \frac{{\sigma_{q}^{2} }}{{\sigma_{q}^{2} + \sigma_{g}^{2} + \sigma_{e}^{2} }}$

^b^Correlation between true breeding values and genetic values explained by QTL detected with StepLMM

^c^Correlation between true breeding values and genetic values excluding QTL detected with StepLMM

^d^Correlation between true breeding values and genomic breeding values estimated with StepLMM

^e^Correlation between true breeding values and genomic breeding values estimated with GBLUP

^f^Regression coefficient of the true on the estimated breeding values with StepLMM

^g^Regression coefficient of the true on the estimated breeding values with GBLUP

### Comparison with other methods

QTLMAS16 datasets have been used to compare different methods of GS (Table 3) and GWAS (Table 4), hence, it is convenient to compare StepLMM with other methods to evaluate its robustness. Here, we selected several methods mentioned in [27] for comparison. Table 3 shows that the accuracy of genomic prediction with StepLMM was the same as that with group least angle shrinkage and selection operator (GLASSO) [28] and sparse group LASSO (sgLASSO) [28] for the second trait. StepLMM predicted breeding values more accurately than the other methods for the first and third traits. Overall, StepLMM performed best among all methods.

**Table 3:** Comparison of the genomic prediction accuracy between stepwise linear mixed model (StepLMM) and other methods based on QTLMAS16 data

Method	Trait 1	Trait 2	Trait 3
BayesB	0.79	0.83	0.83
GBLUP	0.74	0.77	0.76
GLASSO^a^	0.79	0.85	0.84
sgLASSO^b^	0.80	0.85	0.82
StepLMM	0.83	0.85	0.85

^a^Group least angle shrinkage and selection operator [28]

^b^Sparse group LASSO [28]

**Table 4:** Comparison of the mapping precision between stepwise linear mixed model (StepLMM) and other methods based on QTLMAS16 data with 50 simulated QTL

Method	Number of false positives				Number of true QTL				Ratio^a^
	Trait1	Trait2	Trait3	Total	Trait1	Trait2	Trait3	Total	
RR_YD^b^	9	15	5	29	8	6	8	22	0.43
GRAMMAR^c^	0	0	0	0	2	3	5	10	1.00
RHM20^d^	1	0	0	1	6	4	7	17	0.94
RF_YD^e^	3	2	0	5	3	3	5	11	0.69
LDLA^f^	3	3	1	7	6	2	5	13	0.65
LA^g^	4	3	1	8	0	1	2	3	0.27
StepLMM	0	0	0	0	5	4	2	11	1.00

^a^Calculated as the ratio of the number of detected true QTL to the number of all detected QTL

^b^Ridge regression on actual yield deviations [29]

^c^Genome-wide rapid association using mixed model and regression [30]

^d^Regional heritability mapping (20 SNPs) [32]

^e^Random forest with yield deviations [39]

^f^Linkage disequilibrium and linkage analysis [31]

^g^Linkage analysis [33]

In QTL mapping (Table 4), ridge regression on yield deviations (RR_YD) [29] detected the largest number of QTL, but also the largest number of false positives, i.e. RR_YD had the highest power and also the highest rates of false positives. StepLMM, as well as GRAMMAR [30], did not identify any false positive QTL for all three traits, but detected one more QTL than GRAMMAR, i.e. both methods had medium mapping power and very low rates of false positives. The combined linkage disequilibrium and linkage analysis method (LDLA) [31] had medium mapping power, but a relatively high rate of false positives. Among all the methods used for QTL mapping, regional heritability mapping with 20 SNPs (RHM20) [32] had a relatively high mapping power and low rate of false positives, while linkage analysis (LA) [33] had the lowest mapping power and a relatively high rate of false positives. If the proportion of true QTL among all detected QTL was used to measure mapping performance, StepLMM and GRAMMAR performed best.

## Discussion

A quantitative trait is controlled both by variants with a large effect and by variants with a small effect. A statistical model that best captures the genetic architecture of a quantitative trait will fit the data of the trait better and provide more accurate estimates of the genetic effect. StepLMM divides the breeding values into QTL and polygenic effects, and fits them with a fixed effect and normally distributed random effect separately, which basically conforms to the genetic architecture of a quantitative trait. StepLMM not only detects variants with a large effect, but also estimates breeding values, hence it is a combination of GWAS and GS. StepLMM is not only an extension, but also a combination of stepwise linear regression and linear mixed regression model. The process of model optimization is also a process of significance test for SNPs in the evaluation model, which avoids determining the threshold values as in traditional association mapping with a single-locus model. The computational burden of StepLMM depends on how many QTL are detected. Compared to other LMM methods, StepLMM needs to estimate variance components only a few times (equal to the number of detected QTL). Because the number of QTL is very small compared to the number of markers, StepLMM has a clear computational advantage. As a multi-locus model, StepLMM can also detect variants with a large effect to improve genomic prediction for species without a genome map, because the stepwise procedure does not depend on either a physical or genetic map. It is useful for many species in aquaculture, for which no genome assembly is available so far.

We found that StepLMM has a high mapping precision and a low rate of false positives and that the balance between these two objectives is good, which is similar to GRAMMAR [30] and regional heritability mapping with 20 SNPs as a region (RHM20) [32]. These three methods can fit the population structure through a realized genetic relationship matrix, which can improve mapping precision and decrease the rate of false positives. A realized genetic relationship matrix constructed with marker data is more accurate than a numerator relationship matrix constructed with pedigree data, and thus improves mapping precision. StepLMM is a multiple-QTL mapping model, which usually has more mapping precision than single-QTL models [16].

Many studies have shown that GBLUP models perform well for most traits in livestock [34, 35], but are not satisfactory for the analysis of simulated data where traits are controlled by a small number of QTL [36, 37]. The results from our analysis on simulated human data showed that accuracy of genomic prediction using GBLUP is equal to about the square root of heritability. An important feature of the simulated human data is that genetic relationships between individuals are very weak. The low prediction accuracy obtained with GBLUP indicates that this method is not sufficiently accurate for genomic prediction in a population with distantly related individuals.

Compared to GBLUP, StepLMM divides breeding values into two parts: major effect and minor effect. Its characteristics contribute to high QTL mapping precision and high genomic prediction accuracy. The accuracy of genomic prediction can be expressed as [10, 38]:

11 $$r_{{g,\hat{g}}} = \sqrt {\frac{{\beta h^{2} }}{{\beta h^{2} + M_{e} /N}}} ,$$

where $\beta = \sigma_{q}^{2} /(\sigma_{q}^{2} + \sigma_{g}^{2} )$, $\sigma_{q}^{2}$ and $\sigma_{g}^{2}$ are the variances explained by QTL and polygenes, respectively. $N$ is the number of phenotypic observations, $M_{e}$ is the effective number of segments in the genome, and $h^{2}$ is the heritability. When more QTL are identified, $\sigma_{q}^{2}$ tends to be high and $\beta$ increases, thus $r_{{g,\hat{g}}}$ increases as well. Simultaneously, when more QTL are identified, $\sigma_{g}^{2}$ tends to be low and the correlations between polygenic effects and TBV become weaker than those of GBLUP. StepLMM showed superiority in both the GWAS and GS, indicating that it can describe the genetic architecture of quantitative traits well, possibly by distinguishing the genes with major effects from those with minor or null effects. Since major and polygenic effects follow different distributions, fitting these effects with different distributions (e.g. StepLMM) is more robust than fitting them with the same distribution (e.g. GBLUP) and consequently improves the genomic prediction accuracy. GS and GWAS are mutually beneficial in StepLMM, since precisely mapped QTL will improve genomic prediction accuracy, while fitting polygenic effects improves GWAS precision. During our analysis, we defined bias as the regression coefficient of the true breeding value on the estimated breeding value and found that the results were sometimes biased (Table 2), although prediction accuracy with StepLMM is high. The degree of bias depended on heritability and power, low heritability and low power indicated a large random error, which led to large bias. How to decrease bias should be studied further.

## Conclusions

A quantitative trait is controlled both by major variants with large effects and by polygenic effects, they are separately fitted with GWAS and GBLUP in StepLMM. GWAS identifies QTL and simultaneously improves genomic prediction accuracy; GBLUP accounts for polygenic effects, it also increases mapping precision and decreases the rate of false positives of GWAS. StepLMM has a high performance in both GWAS and GS and is feasible for agricultural breeding programs and human genetic studies.

## Additional file

**Additional file 1.** R code of StepLMM. The data contains the R function of StepLMM, an example data and users’ guide.
